# Emerging IO checkpoints in gastrointestinal oncology

**DOI:** 10.3389/fimmu.2025.1575713

**Published:** 2025-07-24

**Authors:** Alireza Tojjari, Anwaar Saeed, Ludimila Cavalcante

**Affiliations:** ^1^ Department of Medicine, Division of Hematology & Oncology, University of Pittsburgh Medical Center (UPMC), Pittsburgh, PA, United States; ^2^ UPMC Hillman Cancer Center, Pittsburgh, PA, United States; ^3^ Department of Hematology and Medical Oncology, University of Virginia Comprehensive Cancer Center, Charlottesville, VA, United States

**Keywords:** immune checkpoints, gastrointestinal oncology, TIGIT, GITR, VISTA, STING, TIM-3, cancer immunotherapy

## Abstract

Recent progress in immunotherapy has significantly altered the therapeutic approach for gastrointestinal cancers, which are historically challenging due to their intricate pathologies and unfavorable outcomes. This review emphasizes the growing importance of immune checkpoints like TIGIT, VISTA, GITR, STING, and TIM-3 in the treatment of gastrointestinal oncology. These checkpoints are crucial elements within the tumor microenvironment, presenting new therapeutic possibilities. Studies show that TIGIT and GITR regulate the functions of T cells and NK cells, while the VISTA and STING pathways boost the body’s anti-tumor responses. TIM-3 is linked with T cell fatigue, highlighting its potential as a target to counteract immune evasion mechanisms. Integrating these immune checkpoints with traditional treatments could result in more customized and effective therapeutic approaches. This detailed review seeks to explore the changing field of immune checkpoint research, offering insights from molecular biology to clinical practice, and envisioning a future where advanced treatment methods greatly enhance patient outcomes in GI cancers.

## 1 Introduction

Immunotherapy has revolutionized cancer treatment, bringing new possibilities for patients whose conditions did not respond to traditional methods. This transformation is especially evident in gastrointestinal (GI) cancers. Gastrointestinal cancers remain notoriously resistant to immunotherapy, largely because their dense, immunosuppressive microenvironments and heterogeneous tumor biology blunt the effectiveness of conventional checkpoint inhibitors. T cell immunoreceptor with Ig and ITIM domains (TIGIT), V-domain Ig suppressor of T cell activation (VISTA) blockade, glucocorticoid-induced TNFR-related protein (GITR) agonism, Stimulator of Interferon Genes (STING) pathway activation, and T cell immunoglobulin and mucin-domain containing-3 (TIM-3) inhibition—has revealed strategies to both unleash CD8^+^ T-cell cytotoxicity and dismantle regulatory networks that foster tumor tolerance. By selectively enhancing T-cell receptor signaling, promoting type I interferon responses, and alleviating suppressive cues within the tumor niche, these emerging checkpoints hold promise for overcoming the core hurdles in GI cancer treatment (1) (2).

Enhancing this cadre of immune regulators is the TIM-3, a checkpoint that has emerged as a focal point of interest due to its association with T cell exhaustion and its potential as a cancer therapy target. TIM-3 is expressed on diverse immune cells, including T cells, natural killer cells, and dendritic cells. Its role in regulating immune responses and maintaining immune equilibrium is significant. The interaction between TIM-3 and its ligands, such as galectin-9, leads to the suppression of T-cell functionality, aiding in the immune evasion tactics of tumors. TIM-3 interactions extend beyond galectin-9 to include CEACAM-1, phosphatidylserine (PtdSer), and HMGB1. Recently, sabatolimab, a TIM-3 antibody, received FDA Fast Track designation (May 2021), emphasizing its potential clinical relevance (3). Recent investigations highlight TIM-3’s significance in the context of T cell exhaustion and its correlation with the efficacy of anti-PD-1 therapy, suggesting that targeting TIM-3 could represent a promising strategy in the realm of cancer immunotherapy (4).

This body of research underscores the need for a deep understanding of the tumor microenvironment (TME) and the complex roles played by immune checkpoints, including STING, in GI cancers. The nuanced expression and functional roles of TIGIT, VISTA, GITR,STING, and TIM-3 revealed through advanced genomic and immunological analyses provide a foundation for developing innovative combination therapies designed to overcome the limitations of existing treatments (5, 6).

Current standard immunotherapies for gastrointestinal (GI) cancers primarily involve the use of immune checkpoint inhibitors targeting the PD-1/PD-L1 axis, such as pembrolizumab and nivolumab. These therapies have significantly improved outcomes, especially in subsets of patients with microsatellite instability-high (MSI-H) or mismatch repair-deficient (dMMR) tumors, highlighting the therapeutic potential of immune modulation in GI oncology (1, 7).

As we move forward in the field of cancer immunotherapy, the exploration of TIGIT, VISTA, STING, GITR and TIM-3 within GI cancers expands our arsenal of therapeutic strategies and enhances our understanding of the intricate interactions between cancer and the immune system. This review explicitly aims to answer how targeting emerging checkpoints such as TIGIT, VISTA, GITR, STING, and TIM-3 can improve therapeutic outcomes in gastrointestinal oncology (5, 6, 8).

## 2 Background

Investigating immune checkpoint receptors like TIGIT, VISTA, GITR, TIM-3 and STING stands at the forefront of cancer research, unveiling new pathways to adjust the immune system’s reaction to tumors. These receptors are crucial in managing immune responses and frequently cross-regulate each other, marking them as valuable prospects for treatment strategies. It is essential to grasp the distinct roles and interactions these immune checkpoints have within the immune framework and their significance in the context of cancer treatment. This understanding could pave the way for groundbreaking approaches in immunotherapy, targeting these mechanisms to combat cancer more effectively.

### 2.1 TIGIT

TIGIT, an essential co-inhibitory receptor found on T cells and NK cells, is expressed on CD8^+^ T cells, NK cells, regulatory T cells (Tregs), CD4^+^ T cells, and dendritic cells. Studies have shown that it has an aiding impact on regenerative hyperplasia, indicating that liver regeneration is compromised in its absence *in vivo* (9). This is achieved by suppressing intracellular activities in NK and dendritic cells and curbing the growth and functionality of T cells (10). The engagement of TIGIT with its binding partners, poliovirus receptor (PVR) (CD155), nectin-2 (CD112), and nectin-4 (PVRL4)—plays a pivotal role in modulating immune reactions and the evolution of cancer. CD155, as the primary binding partner of TIGIT, predominantly initiates suppressive signals in the immune system and is linked with unfavorable outcomes in cancer cases (11–13). On the other hand, the interaction between TIGIT and CD226 attenuates immune operations, while nectin-2 and nectin-4 are identified as promising targets in cancer treatment due to their effects on the behavior of tumor cells (14, 15).

The presence of TIGIT on CD8+ T cells and NK cells is associated with a suppressive immune state and the production of cytokines, underscoring its significance in autoimmune diseases and various cancers like acute myeloid leukemia, chronic myelogenous leukemia, lung cancer, and melanoma (16, 17).

**Figure 1:**
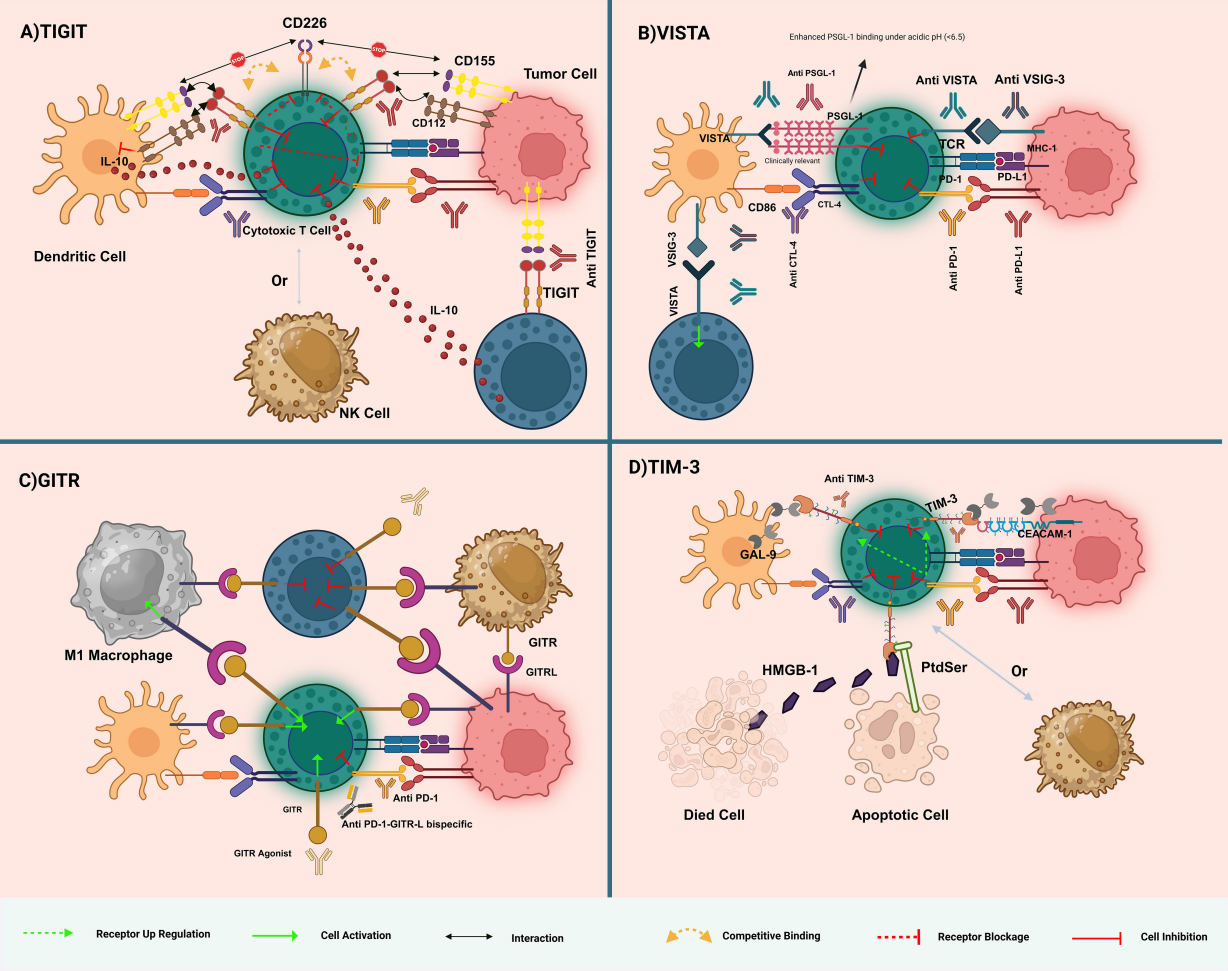
Overview of immune checkpoint interactions and therapeutic targets in immunotherapy. **(A)** TIGIT Pathway: TIGIT competes with CD226 for binding to CD155 on dendritic cells, leading to immune suppression. Anti-TIGIT antibodies block this inhibitory interaction, enhancing T-cell activation by allowing CD226 to bind CD155, thereby boosting anti-tumor immunity. **(B)** VISTA Pathway: VISTA interacts with VSIG-3 and B7 family members to suppress T-cell responses. VISTA binding to PSGL-1 is significantly enhanced under acidic conditions (pH < 6.5), as found in the tumor microenvironment. Anti-VISTA antibodies disrupt this suppression, restoring T-cell function. Combining anti-VISTA with other checkpoint inhibitors (e.g., anti-PD-1, anti-CTLA-4) can further enhance T-cell activation and reduce immune evasion by tumors. **(C)** GITR Pathway: GITR activation by agonistic antibodies or GITRL enhances T-cell cytokine production and immune response. GITR activation also depletes regulatory T cells (Tregs), which suppress immunity. Targeting GITR with bispecific antibodies like anti-PD-1-GITR-L aims to activate effector T cells and reduce Treg-mediated suppression, promoting tumor rejection. **(D)** TIM-3 Pathway: TIM-3 interacts with galectin-9, CEACAM-1, PtdSer, and HMGB1, contributing to T-cell exhaustion. Anti-TIM-3 antibodies block these inhibitory signals, reinvigorating T-cell function and promoting anti-tumor immunity. TIM-3 blockade is particularly effective when combined with PD-1/PD-L1 inhibitors to overcome multiple layers of immune suppression. Dashed arrows indicate reported cross-regulatory loops (e.g., TIGIT–PD-1 co-inhibition). The figure illustrates the dynamic complexity of immune checkpoint pathways, highlighting potential therapeutic strategies in cancer immunotherapy.

However, while targeting TIGIT presents a valuable opportunity in treating blood cancers, the potential for adverse side effects demands vigilant observation of patients. Reports from clinical studies of anti-TIGIT treatments have highlighted immune-related adverse events, emphasizing the importance of a thorough assessment of these therapies (7, 18). The CITYSCAPE study further corroborated these findings, with a significant majority of individuals treated with tiragolumab and atezolizumab reporting adverse immune responses, predominantly skin rashes, in addition to pancreatitis, underactive thyroid, colitis, and diabetes (19). These insights underscore the imperative for medical practitioners to diligently monitor for and mitigate the immunological side effects of TIGIT-targeting treatments(**Figure 1A**).

### 2.2 VISTA

VISTA, or PD-1H, is predominantly expressed on myeloid cells and T-regulatory cells, including CD4+ and Foxp3+ subsets. VISTA is also expressed on activated T cells in specific immune contexts. Its presence on tumor-infiltrating lymphocytes (TILs) and macrophages, juxtaposed with its general absence in most tumor cell types, underpins its complex role in cancer (20). Notably, VISTA has been identified in varying proportions within tumor cells across a spectrum of cancers, such as NSCLC, hepatocellular carcinoma, ovarian and endometrial cancers, melanoma, gastric cancer, and breast cancer (21). Functioning as a negative regulator of T-cell activation, proliferation, and cytokine production, VISTA mainly suppresses CD4+ T-cell-mediated immune responses. This regulatory role is further complicated by the discovery of a fusion protein (VISTA-Ig) that acts as a ligand, illustrating VISTA’s dual function in potentially enhancing proliferation and cytokine production in CD4+ T-cells, thereby indicating its receptor functionality. Moreover, VISTA’s direct influence on the effector functions of myeloid cells underscores the necessity for an in-depth investigation into its multifaceted role in immune regulation, emphasizing its significance as a target in cancer immunotherapy (22, 23).

Adjacent to VISTA’s regulatory mechanisms, VSIG-3, or IGSF11, serves as a ligand for VISTA, implicated in cell adhesion processes and expressed predominantly in human tumor cell lines, testes, and ovaries, with lesser expression noted in the brain and kidneys (24). The interaction between VISTA and VSIG-3 curtails the production of IL-2, IFN cytokines, and various chemokines by activated T-cells and peripheral blood mononuclear cells, delineating its immunosuppressive capacity (25). The overexpression of VSIG-3, correlated with high tumor malignancy and poor prognosis, has been identified in cancers such as colorectal and hepatocellular carcinoma, often in association with PD-L1 and PD-1 expressions (26). Despite acknowledging its role in immunosuppression, the precise mechanisms by which VSIG-3 influences cancer pathogenesis remain elusive, with some reports, such as Johnston et al., challenging the specificity of the VISTA-VSIG-3 interaction (27). Despite acknowledgment of its role in immunosuppression, the precise mechanisms by which VSIG-3 influences cancer pathogenesis remain elusive, with some reports, such as Johnston et al., challenging the specificity of the VISTA-VSIG-3 interaction. Further complicating VISTA’s interaction network are its associations with galectin-9 and PSGL-1, particularly under the acidic conditions characteristic of the tumor microenvironment, which hint at novel therapeutic targets. These interactions, especially with PSGL-1 and galectin-9, illuminate VISTA’s critical position within the immune regulatory framework, advocating for extensive research to elucidate effective immunotherapeutic strategies (28, 29)(**Figure 1B**).

### 2.3 GITR

The GITR, known by aliases TNFRSF18, AITR, or CD357, is a critical member of the tumor necrosis factor receptor superfamily, pivotal in modulating immune responses (30). Initially identified in dexamethasone-treated T cells in mice, GITR is broadly expressed across a spectrum of immune cells, including CD4+ and CD8+ T cells, regulatory T cells (Tregs), natural killer (NK) cells, macrophages, and dendritic cells (31). The interaction between GITR and its ligand, which is present on antigen-presenting cells and various immune cells, plays a fundamental role in a range of immunological functions such as T cell activation, differentiation, survival, regulation of Treg function, and enhancement of effector T cell activities against tumors and infections.

Activation of GITR is instrumental in counteracting Treg-mediated suppression, enhancing effector T cell functions, and promoting anti-tumor immunity. This efficacy positions GITR as a promising target for immunotherapy. Furthermore, the interaction between GITR and its ligand (GITRL) on antigen-presenting cells highlights the complex signaling pathways mediated by GITR that regulate the balance between Treg and effector T cell activities (32).

GITR’s role extends beyond T cells, influencing NK cells, dendritic cells (DCs), and macrophages, indicating its extensive impact on the immune system. The therapeutic potential of modulating GITR-GITRL interactions is explored through agonistic approaches that enhance tumor immune responses and strategies to dampen excessive immune reactions in autoimmune and inflammatory diseases (33). This dual approach to manipulating GITR pathways underscores its significance in developing immunotherapeutic strategies and managing various immune-related conditions (**Figure 1C**).

### 2.4 TIM-3

Tim-3 was originally discovered as a cell surface molecule preferentially expressed on interferon-gamma (IFN-γ)-producing CD4+ Th1 and CD8+ T cells. It belongs to the TIM gene family, which are found in syntenic chromosomal regions linked with both allergy and autoimmune diseases (34). Tim-3 attracted interest when it was discovered as a T-cell inhibitory receptor, and the concept was further supported by the studies showing that *in vivo* administration of monoclonal antibody specific to Tim-3 (mAbs) ameliorated or exacerbated disease in models of autoimmunity. With the exception of lacking a canonical inhibitory signaling motif in its cytoplasmic tail, Tim-3 is considered an immune checkpoint because of the negative regulatory roles it performs (35). This association with diseases driven by hyperactive immune cells sets Tim-3 in place as an important modulator of immune response. Clinical trials with Tim-3 antibodies, either alone or in combination with another treatment, have yielded promising results in different tumors, thus supporting their potentiality as an agent within cancer immunotherapy (**Figure 1D**).

### 2.5 STING

**Figure 2:**
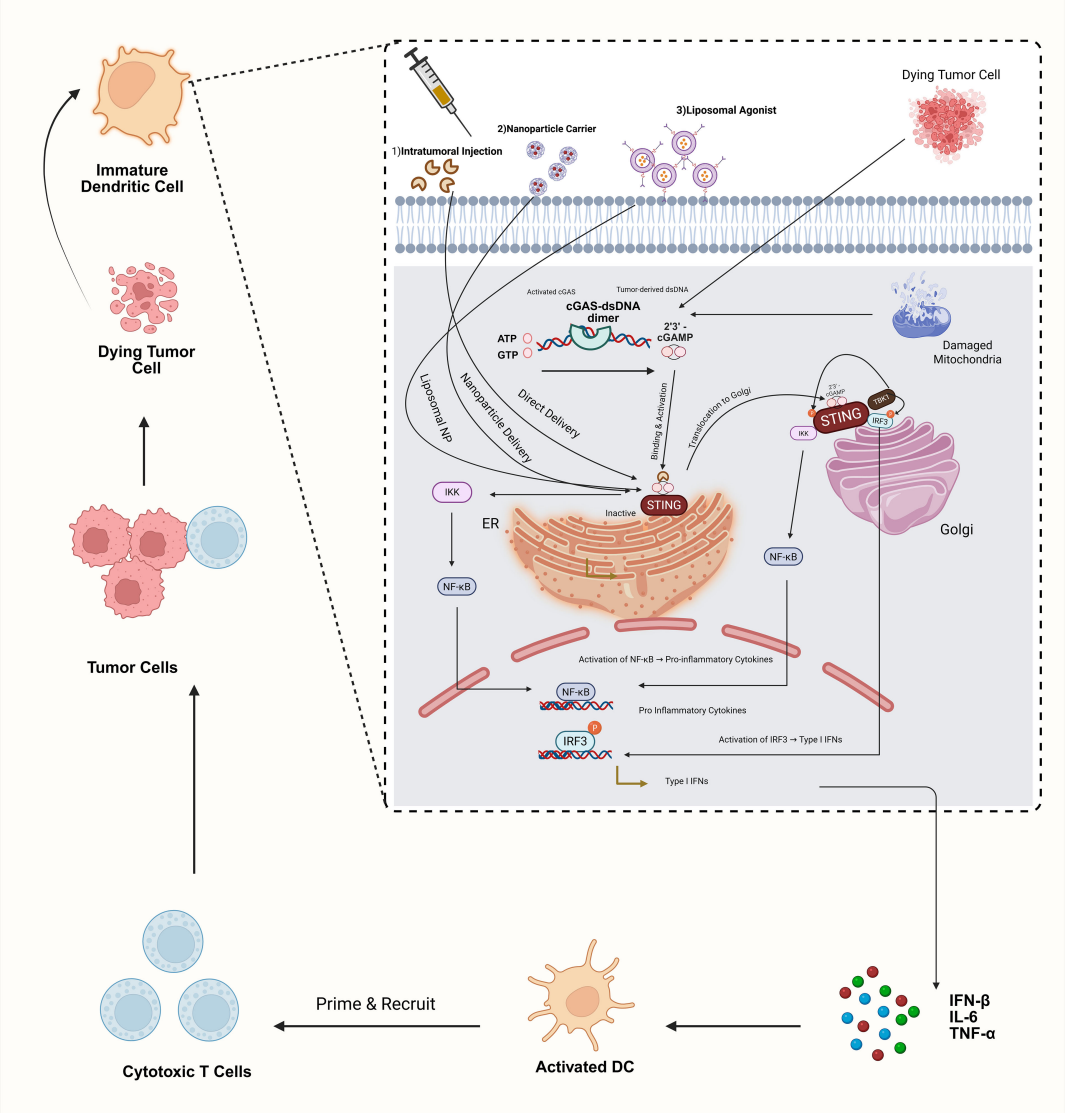
STING pathway activation with agonist delivery strategies. This schematic illustrates activation of the cGAS–STING axis in dendritic cells by (1) tumor-derived cytosolic dsDNA via cGAS conversion to 2′3′-cGAMP and (2) synthetic STING agonists delivered intratumorally, in polymeric nanoparticles, or in liposomal vesicles. Agonist binding induces STING translocation to the Golgi, recruitment of TBK1 and IKK, phosphorylation of IRF3, and NF-κB activation. The resulting type I interferon and pro-inflammatory cytokine production drive DC maturation and CD8^+^ T-cell priming. Clinical translation remains challenging due to inefficient agonist delivery and systemic toxicity.

The STING pathway plays a pivotal role in the body’s innate and cancer immune responses by detecting tumor-derived DNA. This detection triggers the activation of interferon genes, thereby mobilizing antitumor immunity. Advances in drug delivery technologies have facilitated the systemic administration of STING agonists, demonstrating a promising capacity for eliminating tumors in preclinical experiments (36). The innovation in the design of novel STING agonists, including non-cyclic dinucleotide (CDN) molecules such as amidobenzimidazole (ABZI) and its analogs, addresses prior challenges concerning stability and cellular uptake (37, 38). These breakthroughs herald a new era in the application of STING agonist immunotherapy for cancer, offering a comprehensive strategy for managing the disease (**Figure 2**).

## 3 Methods

A systematic literature search was conducted using PubMed, Web of Science, and ClinicalTrials.gov databases from inception until April 2025. Inclusion criteria involved studies on TIGIT, VISTA, GITR, STING, and TIM-3 in gastrointestinal cancers. Exclusion criteria included non-English articles, reviews without original data, and animal studies without clinical relevance. We additionally performed a formal quality assessment of all included studies using the Cochrane Risk-of-Bias tool (Higgins 2011) to evaluate methodological rigor and potential sources of bias (39).

## 4 Preclinical insights into cancer immunotherapy: focusing on TIGIT, VISTA, GITR, STING, and TIM-3 pathways

In the vanguard of oncological research, particularly within the immunotherapeutic domain, a series of foundational preclinical investigations have cast a spotlight on the TIGIT, VISTA, GITR, STING and TIM-3 signaling conduits. These studies delineate these conduits as quintessential targets for the genesis of groundbreaking therapeutic modalities aimed at potentiating the immune apparatus’s prowess in eradicating malignancy.

### 4.1 TIGIT

#### 4.1.1 Mechanism

TIGIT functions as an inhibitory receptor primarily expressed on CD8^+^ T cells, CD4^+^ T cells, Tregs, and NK cells. It competes with the co-stimulatory receptor CD226 (DNAM-1) for binding to shared ligands CD155 (PVR) and CD112 (PVRL2) on antigen-presenting cells and tumor cells. Upon engagement, TIGIT transmits an intracellular inhibitory signal via its ITIM and ITT-like motifs, leading to decreased AKT phosphorylation, suppression of TCR signalling, and reduced secretion of IL-2, IFN-γ, and TNF-α. It also enhances Treg suppressive capacity while impairing NK cell cytotoxicity and promoting immune exhaustion. TIGIT signalling thus establishes an immunosuppressive microenvironment favorable for tumour survival and metastasis (40) (41–44).

#### 4.1.2 Preclinical evidence

Murine models of head and neck, lung, and colorectal cancers have shown TIGIT upregulation in CD8+ and CD4+ tumor-infiltrating lymphocytes (TILs), often co-expressed with PD-1 and LAG-3 (45–47) TIGIT-blocking antibodies like EOS-448 and bispecific agents such as D3L-002 significantly enhanced T and NK cell-mediated antitumor immunity, particularly when combined with PD-1/PD-L1 inhibitors (48–50). Ex vivo models using GI cancer specimens showed TIGIT antagonists improved cytotoxic T cell function and modulated regulatory T cell suppressive markers (6, 8, 51, 52). Importantly, TIGIT blockade also synergizes with dendritic cell–T cell crosstalk mechanisms via IFN-γ and IL-12 signaling (53).

#### 4.1.3 Model limitations

Mouse models often lack the complexity of human TME, particularly in TIGIT’s expression on Tregs and its interplay with other checkpoints. Differences in cytokine milieus and receptor-ligand affinities may limit the translatability of these results to human tumors (54, 57).

Phase III trials SKYSCRAPER-01 (tiragolumab + atezolizumab), SKYSCRAPER-06, and KeyVibe-008 (vibostolimab) failed due to lack of efficacy, diminishing expectations for TIGIT-targeted therapies (54–56).

### 4.2 VISTA

#### 4.2.1 Mechanism

VISTA, also known as PD-1H, is an inhibitory checkpoint receptor predominantly expressed on myeloid-derived suppressor cells (MDSCs), tumor-associated macrophages (TAMs), and Tregs. Unlike other checkpoints, VISTA can function as both a ligand and a receptor. When acting as a ligand, it binds to PSGL-1 and VSIG-3 on T cells, especially under acidic pH conditions of the tumor microenvironment, delivering a suppressive signal that reduces T cell proliferation and cytokine production. Notably, VISTA binding to PSGL-1 is significantly enhanced under acidic conditions typical of the tumor microenvironment, underscoring its dual inhibitory and stimulatory capacities dependent on local context (27).

As a receptor, VISTA directly transmits inhibitory signals into myeloid cells, reducing their antigen presentation capabilities and skewing them toward a tolerogenic phenotype. This dual functionality enables tumors to exploit VISTA to blunt both innate and adaptive immune responses (23, 57, 58).

#### 4.2.2 Preclinical evidence

In murine models of colorectal and pancreatic cancer, VISTA blockade led to increased CD8^+^ T cell infiltration and enhanced IFN-γ production. Combination therapy with anti-VISTA and anti-PD-1 antibodies showed superior tumor control compared to monotherapy, suggesting synergistic immunomodulatory effects (59, 60).

#### 4.2.3 Model limitations

VISTA’s expression profile and immune functions differ significantly between species. In mice, VISTA is predominantly expressed on granulocytic MDSCs, while in humans it is more prominent on monocytic subsets. Additionally, the lack of homologous antibodies that mimic human VISTA-targeting compounds limits the translatability of murine findings. Preclinical models often fail to account for the dynamic regulation of VISTA in chronic inflammation and its context-dependent role in tolerance versus activation (21, 61).

### 4.3 GITR

#### 4.3.1 Mechanism

GITR is a co-stimulatory immune receptor found constitutively on Tregs and upregulated on activated CD4^+^ and CD8^+^ T cells. Its ligand, GITRL, is expressed on APCs such as dendritic cells and macrophages. Upon ligand engagement, GITR activates the NF-κB and MAPK pathways through TRAF2 and TRAF5 signaling adaptors, promoting survival, proliferation, and effector functions in CD8^+^ T cells and Th1/Th17 subsets. Simultaneously, GITR ligation impairs Treg suppressive capacity by destabilizing FOXP3 expression and disrupting IL-10/TGF-β signaling loops. This dual effect enhances overall immune activation, making GITR a compelling target for restoring anti-tumor immunity, especially in immunosuppressive microenvironments (31, 62, 63). GITR agonist monotherapies have largely failed clinically due to dose-limiting immune toxicity and insufficient stimulation of anti-tumor immunity (64).

#### 4.3.2 Preclinical evidence

GITR agonists have demonstrated synergy with PD-1 and LAG-3 inhibitors in murine models of melanoma and NSCLC, leading to improved tumor control and survival (62, 63, 65, 66). In GI tumor ex vivo models, GITR agonists selectively enhanced cytotoxic CD8^+^ T cell responses (6, 8, 66). Despite promising preclinical synergy, early-phase clinical monotherapy trials of GITR agonists (e.g., REGN6569 + cemiplimab) have encountered dose-limiting toxicities and underwhelming antitumor activity in solid tumors (67).

#### 4.3.3 Model limitations

Rodent Tregs differ functionally from human Tregs in response to GITR agonism, necessitating caution in interpretation (68).Moreover, dose-dependent overstimulation of the immune system can trigger paradoxical immune suppression or autoimmune-like pathologies in some preclinical models. Additionally, the translation of efficacy from murine systems to humans has been inconsistent due to interspecies variation in GITR/GITRL signaling cascades (63).

### 4.4 STING

#### 4.4.1 Mechanism

STING (Stimulator of Interferon Genes) is a cytosolic adaptor protein that senses the presence of aberrant cytosolic DNA, either through direct binding to cyclic dinucleotides (CDNs) like cGAMP or downstream of DNA sensors like cGAS. Upon activation, STING translocates from the ER to the Golgi, initiating a cascade involving TBK1-mediated phosphorylation of IRF3, leading to transcription of type I interferons (IFN-α/β) and proinflammatory cytokines. This immune activation recruits dendritic cells and primes cytotoxic T lymphocytes against tumor antigens. In the TME, STING activation enhances antigen presentation, reverses immune suppression, and can modulate stromal components like CAFs, reducing desmoplasia and improving immune cell infiltration (36, 69) (70, 71). Clinical application of STING agonists remains challenging due to delivery limitations and systemic toxicity, complicating their translation into effective treatments (72).

#### 4.4.2 Preclinical evidence

STING agonists (e.g., ADU-S100) in combination with TLR9 ligands enhanced immune infiltration and reduced CAFs in colon carcinoma models, improving survival. Activation of STING pathways also boosted NK cell responses and proinflammatory cytokine levels in adjacent spleen tissues (69, 70).

#### 4.4.3 Model limitations

Murine STING exhibits different binding affinities and downstream activation profiles compared to human STING, which can lead to overestimation of therapeutic efficacy in preclinical. Additionally, several human tumors show low baseline STING expression or have mutations in STING-related pathways, which are not recapitulated in mouse models (71). However, systemic STING agonists continue to be hampered by cytokine-release toxicities and dose-limiting myelitis, as reported in early MIW815 (ADU-S100) trials (37).

### 4.5 TIM-3

#### 4.5.1 Mechanism

TIM-3 (T cell immunoglobulin and mucin-domain containing-3) is an inhibitory receptor expressed on exhausted CD8^+^ T cells, Tregs, NK cells, and dendritic cells. It binds to multiple ligands: Galectin-9 (induces apoptosis of effector T cells), phosphatidylserine (mediates clearance of apoptotic cells), HMGB1 (inhibits innate immune activation), and CEACAM1 (regulates tolerance and exhaustion). Upon ligand binding, TIM-3 disrupts TCR signaling by interacting with Bat3 and Fyn, leading to suppression of Th1 cytokines and CTL activity. In dendritic cells, TIM-3 downregulates nucleic acid sensing, reducing IFN production. TIM-3 also maintains Treg stability and suppresses inflammation, making it a key regulator of T cell exhaustion and immune dysfunction in chronic tumors (73–76).

#### 4.5.2 Preclinical evidence

Preclinical studies using radiolabeled anti-TIM-3 antibodies have demonstrated selective uptake in colon and breast cancer mouse models. Co-blockade of TIM-3 with PD-1 restored CD8^+^ T cell function and suppressed tumor growth. TIM-3 targeting has also shown promise in selectively eliminating leukemia stem cells (76).

#### 4.5.3 Model limitations

TIM-3 exhibits heterogeneity in expression across immune cell subsets and cancer types, making it difficult to identify predictive biomarkers for response. Moreover, ligand-binding affinity and downstream signalling differ between mice and humans due to species-specific receptor-ligand interactions (77). Additionally, monoclonal antibodies targeting TIM-3 may bind distinct epitopes in murine versus human systems, affecting pharmacodynamics and efficacy. These discrepancies necessitate validation in humanized or patient-derived models before clinical translation (77).

These preclinical ventures provide critical insights into the intricate mechanisms underlying immunological evasion and identify novel therapeutic targets. The orchestration of therapies derived from these insights is poised to revolutionize oncological care, enhancing the immunological arsenal against neoplastic entities. **Table 1** provides a summary of the preclinical evaluation of immunotherapy checkpoints in gastrointestinal cancer models.

**Table 1:** Summary of preclinical evaluation of immunotherapy checkpoints in gastrointestinal cancer models.

Target	Primary mechanism	GI models tested	Outcome	Model limitations
TIGIT	Inhibits T and NK cells via CD155	CRC, gastric, ex vivo tumor slices	↑CD8+, ↑NK response (w/PD-1)	Poorly reflective cytokine milieu
VISTA	Suppresses T cell activation via MDSCs	Pancreatic, gastric	↑CD8+ infiltration, ↓TME suppression	Mouse–human differences in expression
GITR	Enhances T effector, suppresses Tregs	Colorectal, ex vivo GI	Selective CD8+ activation	Variable Treg biology
STING	Triggers type I IFN, DC maturation	Colon carcinoma	↓CAF, ↑IFN cytokines	Species-specific STING activity
TIM-3	Regulates T cell exhaustion	Colon (murine), CRC slices	↑T cell reinvigoration (combo PD-1)	Epitope binding divergence

This table summarizes five key immune‐regulatory targets—TIGIT, VISTA, GITR, STING, and TIM-3—detailing their primary mechanisms of action, the specific GI tumor models in which they were tested, observed immunological outcomes, and noted limitations of each model. Abbreviations: CRC, colorectal carcinoma; ex vivo, studies performed on tumor slices outside the organism; CAF, cancer-associated fibroblast; DC, dendritic cell; IFN, interferon; MDSCs, myeloid-derived suppressor cells; NK, natural killer; PD-1, programmed cell death protein-1; TME, tumor microenvironment; Tregs, regulatory T cells.

## 5 Crosstalk, synergy and antagonism between emerging checkpoints

### 5.1 Crosstalk between TIGIT and the PD-1 axis

Functionally exhausted CD8^+^ TILs frequently co-express TIGIT and PD-1; dual blockade re-invigorates proliferation and cytokine release more potently than either antibody alone, as shown in murine head-and-neck and colorectal models and in ex-vivo GI-cancer slices where TIGIT^+^/PD-1^+^ T cells dominate (8, 78, 79).

### 5.2 GITR–TIGIT counter-regulation

GITR agonism destabilises FOXP3 in TIGIT^high Tregs, indirectly relieving TIGIT-mediated suppression of effector CD8^+^ cells, while TIGIT antagonists broaden the response spectrum to include dysfunctional CD8^+^ and TFH-like subsets (80, 81).

### 5.3 VISTA and PD-1 non-redundancy

In colorectal and pancreatic models, VISTA blockade heightens IFN-γ production, and the combination with anti-PD-1 produces superior tumour control versus monotherapy, reflecting parallel, non-overlapping immunosuppressive circuits (60, 82).

### 5.4 STING activation amplifies checkpoint inhibition

STING agonists up-regulate CXCL10 and CCL5, enhancing dendritic-cell priming and sensitising “cold” MSS-CRC and HCC to PD-1 blockade (83).

### 5.5 TIM-3 adaptive up-regulation post PD-1/TIGIT therapy

Preclinical data show compensatory TIM-3 expression after PD-1 ± TIGIT inhibition, supporting bispecific TIM-3/PD-1 antibodies now in phase I (84).

**Figure 3:**
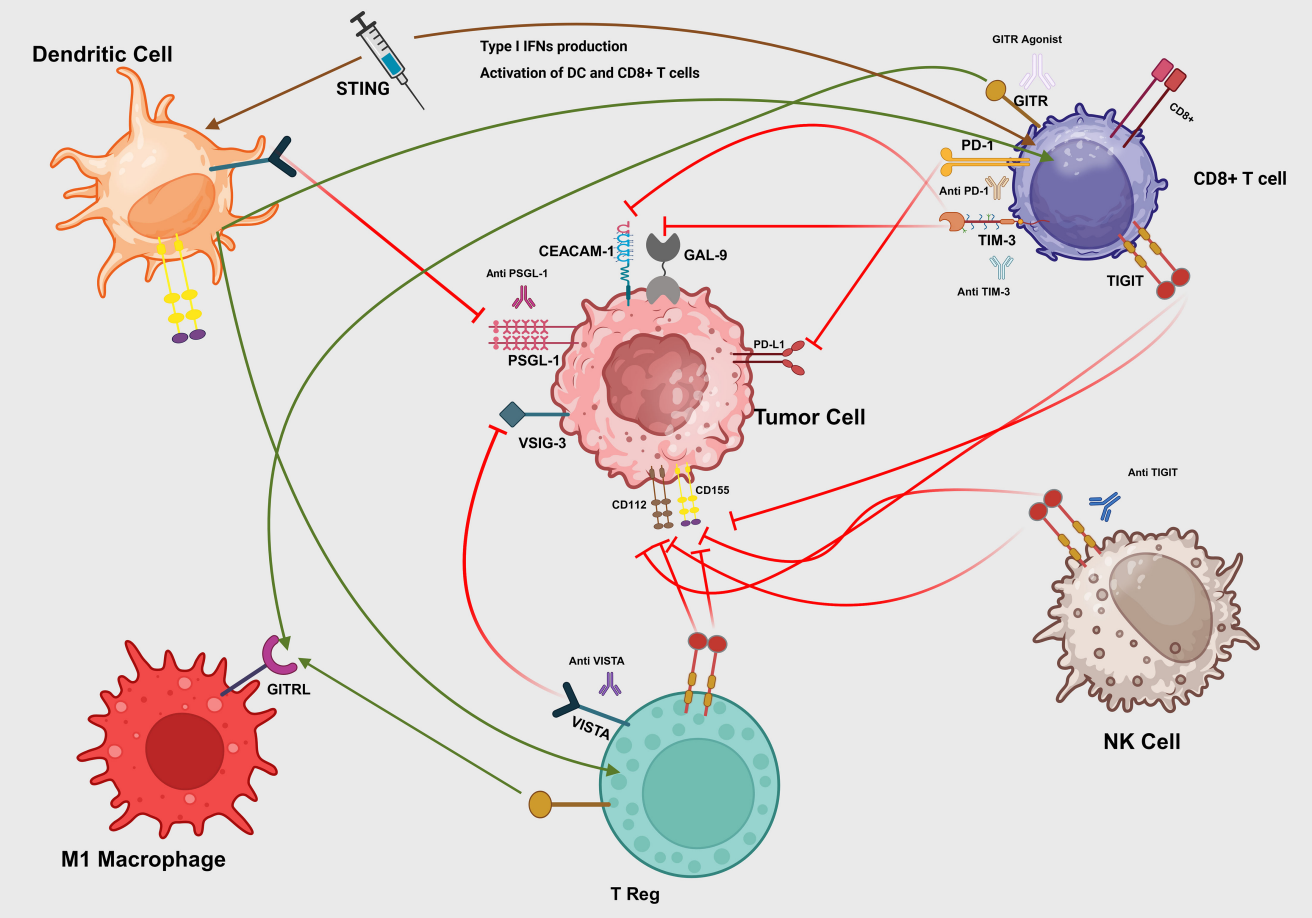
Interactions of immune checkpoints in the tumor microenvironment. A synthetic STING agonist (blue syringe) binds to the STING adaptor on immature dendritic cells (DCs), triggering production of type I interferons (IFN-β) that activate both DCs and CD8^+^ T cells (brown arrows). Mature DCs present tumor antigens and co-stimulatory signals to CD8^+^ T cells (green arrow), priming cytotoxic responses. Tumor-expressed PD-L1 engages PD-1 on CD8^+^ T cells (red T-bar), dampening TCR signaling. Galectin-9 (GAL-9) and CEACAM-1 on tumor cells bind TIM-3 on CD8^+^ T cells (red T-bars), driving exhaustion. Tumor ligands CD155 and CD112 engage TIGIT on NK cells (red T-bar), inhibiting cytotoxicity, which can be blocked by anti-TIGIT antibody. VSIG-3 and PSGL-1 on tumor cells interact with VISTA on Tregs and DCs (red T-bars), enforcing local immunosuppression, while VISTA blockade (green arrow) restores immune activation. M1 macrophage–expressed GITRL binds GITR on Tregs (green arrow), reinforcing suppression, but a GITR agonist antibody can convert this into co-stimulation on effector T cells. Therapeutic antibodies (anti-PD-1, anti-TIM-3, anti-VISTA, anti-PSGL-1) intercept their respective inhibitory axes to reinvigorate DC, T cell, and NK cell functions.

**Figure 3** shows a schematic of these checkpoint interactions and STING-mediated activation in the tumor microenvironment.

## 6 Integration and advancements in immuno-oncology clinical research

### 6.1 Insightful developments in targeting the TIGIT pathway

A pioneering investigation in Japan has showcased the potential of a novel therapeutic combination targeting advanced solid tumors. This approach, employing tiragolumab in conjunction with atezolizumab, has been characterized by its commendable safety profile, opening avenues for further large-scale evaluations. Grade ≥3 treatment-related AEs occurred in 15% of patients, chiefly hypertension and pruritus, and were comparable to atezolizumab-based historical controls (85). The consistency of therapeutic outcomes across various patient profiles underscores the promise of this regimen in future comprehensive trials aimed at establishing its efficacy (86). In line with these advancements, the MORPHEUS-EC trial is a phase Ib/II open-label, randomized study evaluating the efficacy and safety of the combination of tiragolumab (tira) and atezolizumab (atezo) with chemotherapy as a first-line treatment for patients with esophageal cancer. This trial aims to explore the potential benefits of adding immune checkpoint inhibitors to standard chemotherapy regimens, potentially improving patient outcomes in esophageal cancer (87). In this gastric cohort, domvanalimab + zimberelimab + FOLFOX produced an objective response rates (ORR) of 59% (69% in PD-L1-high patients) with a 12-month PFS rate of 58% (88).

Similarly, the MORPHEUS-Liver study focuses on a phase Ib/II randomized evaluation of tira, atezo, and bevacizumab in patients with unresectable, locally advanced, or metastatic hepatocellular carcinoma (uHCC). The primary goal of this study is to determine the efficacy and safety of this combination therapy in enhancing anti-tumor immune responses and improving overall survival rates in patients with advanced liver cancer (89). Across upper- and lower-GI malignancies, TIGIT- or TIM-3–directed combinations are now yielding a consistent response pattern. In PD-L1–high oesophageal and gastric cancers, ORR cluster around 55-70%, with 12-month progression-free survival (PFS) ≥ 50% as seen in MORPHEUS-EC and EDGE-Gastric (87, 90). The regimen’s safety mirrored PD-1 + chemo standards; most common any-grade AEs were neutropenia (61%), nausea (59%), and anemia (29%), with grade ≥3 immune-related events ≤ 2% (88).Hepatocellular carcinoma shows more modest activity (ORR ≈ 40%, median PFS ≈ 7 months), yet still clearly superior to historical PD-1 monotherapy benchmarks (85). By contrast, refractory colorectal cohorts seldom exceed single-digit ORR regardless of checkpoint targeted, mirroring the immunologically ‘cold’ micro-environment of most MSS CRC. This gradient underscores a tumour-intrinsic hierarchy of TIGIT-axis sensitivity across the GI spectrum and guides trial stratification moving forward.

### 6.2 Enhancing immunotherapy efficacy through strategic combinations

The collaboration of vibostolimab with pembrolizumab represents a significant stride in immunotherapy, particularly for NSCLC. This regimen’s ability to produce positive outcomes, particularly in patients new to anti-PD-1/PD-L1 therapy, positions it as a potentially transformative option in cancer treatment. The nuanced effectiveness in different patient subsets highlights the importance of continued exploration and optimization of such therapeutic strategies (7).

### 6.3 Reevaluating GITR’s influence on gastric cancer immunity

The intricate dynamics of GITR within the gastric cancer immune environment suggest a pivotal yet complex role in shaping patient prognosis. The association of GITR expression with a more suppressive tumor microenvironment invites a rethinking of therapeutic approaches, advocating for personalized strategies that consider the multifaceted roles of immune checkpoints. This perspective encourages a deeper examination of GITR’s potential as a therapeutic target, aiming for a more targeted and effective cancer immunotherapy (91).

### 6.4 Prognostic implications of VISTA in colorectal cancer

The relationship between VISTA expression and colorectal cancer outcomes offers new insights into the prognostic landscape of this disease. The correlation of high VISTA levels with several markers of better prognosis highlights its utility as a therapeutic target and a guide for clinical decision-making. This understanding enriches the dialogue on immunotherapeutic innovations and their potential to redefine treatment paradigms (92).

### 6.5 Synergistic frontiers: unveiling the potential of sabatolimab and spartalizumab in advanced solid tumors

In a pioneering phase I/Ib clinical trial, Curigliano et al. explored the combination of Sabatolimab, a TIM-3 antibody, received FDA Fast Track designation in May 2024 and entered Phase II studies by June 2024, with preliminary safety data reported at ASCO24 (93). This study enrolled 219 patients, revealing a tolerable safety profile predominantly characterized by fatigue and identifying an optimal phase II dose for further investigation. Dose-escalation showed ≤ 15% grade ≥3 TRAEs, predominantly fatigue, and no dose-limiting immune-mediated toxicity (94). Notably, the combination therapy elicited partial responses in a subset of patients across various solid tumors, including colorectal cancer and NSCLC, with responses lasting between 12 to 27 months. These findings underscore the potential of targeting the TIM-3 and PD-1 pathways simultaneously, offering a new horizon for patients previously unresponsive to conventional treatments and signaling a significant step forward in the realm of immunotherapy. The trial’s insights into the synergistic efficacy of sabatolimab and spartalizumab pave the way for future explorations into combination therapies, heralding a promising direction for personalized cancer treatment strategie (93). **Table 2** summarises the ongoing trials of TIGIT, VISTA, GITR, and STING.

**Table 2:** Ongoing trials of TIGIT, VISTA, GITR and STING^*^.

NCT number	Title	Phase	Enrollment	Sponsor	Last update	Organ/System/Cancer type	Primary end date	Preliminary results
TIGIT								
NCT05607563	A Study of PM1009 (Anti-TIGIT/PVRIG) in Patients With Advanced Tumours	Phase 1	54	Biotheus Inc.	2023-02-08	Advanced Tumor	Nov 2023	Well-tolerated up to 1200 mg with signs of antitumor activity (95).
NCT05082610	A Study of HMBD-002, a Monoclonal Antibody Targeting VISTA, as Monotherapy and Combined With Pembrolizumab	Phase 1	313	Hummingbird Bioscience	2025-04-18	Various Solid Tumors (e.g., Non-Small Cell Lung Cancer, Triple Negative Breast Cancer)	Nov 2024	No results posted yet.
NCT05329766	A Safety and Efficacy Study of Treatment Combinations With and Without Chemotherapy in Adult Participants With Advanced Upper Gastrointestinal Tract Malignancies (EDGE-Gastric)	Phase 2	360	Gilead/Arcus	2022-06-10	Gastric, Gastroesophageal Junction, Esophageal Cancer	Sep 2026	ORR 59% overall (80% in PD-L1-high, 46% in PD-L1-low) with 2 CRs; 6-month PFS rate 77%; median PFS not reached (88).
NCT04262856	Study to Evaluate Monotherapy and Combination Immunotherapies in Participants With PD-L1 Positive Non-small Cell Lung Cancer (ARC-7)	Phase 2	151	Arcus	2020-05-28	Non-Small Cell Lung Cancer	Feb 2025	Interim analysis: domvanalimab + zimberelimab arms showed clinically meaningful improvements in ORR and PFS compared with zimberelimab alone (doublet median PFS ~9.3 mo vs 5.4 mo control; HR ≈0.51) (96).
NCT04294810	A Study of Tiragolumab in Combination With Atezolizumab Compared With Placebo in Combination With Atezolizumab in Patients With Previously Untreated Locally Advanced Unresectable or Metastatic PD-L1-Selected Non-Small Cell Lung Cancer (SKYSCRAPER-01)	Phase 3	620	Hoffmann-La Roche	2020-03-04	Non-Small Cell Lung Cancer.	Feb 2025	Did not meet co-primary end points: no statistically significant improvement in PFS (7.0 mo vs 5.6 mo; HR 0.78; P = .02, α <.001 required) and no OS benefit (median OS 23.1 mo vs 16.9 mo; HR 0.87; P = .22) (54).
NCT04540211	A Study of Atezolizumab Plus Tiragolumab in Combination With Paclitaxel and Cisplatin Compared With Paclitaxel and Cisplatin as First-Line Treatment in Participants With Unresectable Locally Advanced, Unresectable Recurrent, or Metastatic Esophageal Carcinoma (SKYSCRAPER-08)	Phase 3	461	Hoffmann-La Roche	2020-10-30	Esophageal Squamous Cell Carcinoma	Feb 2023	ORR 59.7% (11.5% CR) vs 45.5% (3.2% CR) control; median DOR 7.1 mo vs 4.3 mo; PFS and OS improvements observed across subgroups (97).
NCT06109779	Rilvegostomig + Chemotherapy as Adjuvant Therapy for Biliary Tract Cancer After Resection (ARTEMIDE-Biliary01)	Phase 3	750	AstraZeneca	2023-12-04	Biliary Tract Cancer	June 2029	No results posted yet.
NCT05568095	STAR-221: Domvanalimab + Zimberelimab + Chemotherapy vs Nivolumab + Chemotherapy in first-line metastatic upper GI adenocarcinomas	Phase 3	1040	Arcus Biosciences/Gilead	2025-06-03	Upper GI adenocarcinoma	Feb 2027	No results posted yet.
VISTA								
NCT04836507	Study of Efficacy and Safety of CRC01 in Adult Large B-cell Lymphoma Patients	Phase 1/2	91	Curocell Inc.	2021-05-03	Lymphoma	May 2023	Anbalcabtagene autoleucel (CRC01) demonstrated a CR 82% (9/11 patients in dose escalation; median follow-up >12 mo in 2 patients) (98).
NCT03198052	GPC3/Mesothelin/Claudin18.2/GUCY2C/B7-H3/PSCA/PSMA/MUC1/TGFβ/HER2/Lewis-Y/AXL/EGFR-CAR-T Cells Against Cancers	Phase 1	30	Second Affiliated Hospital of Guangzhou Medical University	2023-02-14	Cancer/Immunotherapy	Aug 2024	No results posted yet.
NCT05864144	A Study of SNS-101 (Anti VISTA) Monotherapy and in Combination With Cemiplimab in Patients With Advanced Solid Tumors	Phase 1/Phase 2	169	Sensei Biotherapeutics, Inc.	January 17, 2024	Various Solid Tumors (e.g., Breast, Lung, Prostate, Melanoma)	June 2027	No results posted yet.
NCT04305054	Substudy 02B: Safety and Efficacy of Pembrolizumab in Combination With Investigational Agents or Pembrolizumab Alone in Participants With First Line (1L) Advanced Melanoma	Phase 1/2	315	Merck Sharp & Dohme LLC	2024-02-05	Melanoma	April 2030	No results posted yet (ongoing basket study).
NCT05102214	HLX301 (TIGIT×PDL1 Bispecific) in Patients With Locally Advanced or Metastatic Solid Tumors	Phase 1/2	150	Shanghai Henlius Biotech	2023-08-08	Solid Tumors	Sep 2023	Acceptable safety profile with preliminary anti-tumor activity (tumor reductions observed in multiple dose cohorts) (99).
NCT04354246	COM902 (A TIGIT Inhibitor) in Subjects With Advanced Malignancies	Phase 1	110	Compugen Ltd	2023-08-15	Advanced Malignancies	Dec 2023	No results posted yet.
NCT05708950	A Clinical Trial of KVA12123 Treatment Alone and in Combination With Pembrolizumab In Advanced Solid Tumors (VISTA-101)	Phase 1/2	314	Kineta Inc	2023-03-03	Advanced Solid Tumors	June 2024	No results posted yet.
GITR								
NCT04465487	Study of REGN6569 and Cemiplimab in Adult Patients With Advanced Solid Tumor Malignancies	Phase 1	85	Regeneron Pharmaceuticals	2024-01-23	Squamous Cell Carcinoma of Head and Neck	April 2025	ORR 27% (4/15 patients with PR) in naïve cohort; 2 ongoing PRs observed in dose escalation (67).
NCT05017688	Prospective Interventional Study Exploring the Microbiota Recolonization in SR-GvHD Patients Receiving MaaT013	Not Applicable	40	MaaT Pharma	2023-10-11	Intestinal GVHD, Steroid Refractory GVHD	June 2024	No results posted yet.
	Anti-GITR/​Anti-PD1/​Stereotactic Radiosurgery, in Recurrent Glioblastoma	Phase 2	39	University of Pennsylvania	2020-06-23	Glioblastoma	Sep 2022	Did not demonstrate efficacy when given with FSRT (no significant tumor regressions in recurrent GBM) (100).
TIM-3								
NCT05738980	Prevention of Postoperative Recurrence of Hepatocellular Carcinoma by Blocking RAK Cells	Not Applicable	88	Beijing Hospital	February 22 2023	Hepatocellular Carcinoma	Dec 2025	No results posted yet.
NCT03680508	TSR-022 (Anti-TIM-3 Antibody) and TSR-042 (Anti-PD-1 Antibody) in Patients With Liver Cancer	Phase 2	42	Diwakar Davar, Tesaro Inc.,…	October 23 2023	Adult Primary Liver Cancer	Oct 2024	No results posted yet.
NCT06125652	Anti Tim-3/CD123 CAR-T Cell Therapy in AML Refractory	Phase 1/2	20	Beijing Hospital	November 9, 2023	Acute Myeloid Leukemia Refractory	Jan 2026	No results posted yet.
NCT04139902	Neoadjuvant PD-1 Inhibitor Dostarlimab vs. Tim-3 Inhibitor in Melanoma	Phase 2	56	Diwakar Davar, Tesaro Inc.	June 2, 2023	Melanoma Stage III & IV	Jan 2025	No results posted yet.
NCT03961971	Trial of Anti-Tim-3 in Combination With Anti-PD-1 and SRS in Recurrent GBM	Phase 1	16	Johns Hopkins	February 2020	Glioblastoma Multiform	Nov 2022	No results posted yet.
NCT04370704	Study of Combination Therapy With INCMGA00012 (Anti-PD-1), INCAGN02385 (Anti-LAG-3), and INCAGN02390 (Anti-TIM-3) in Participants With Select Advanced Malignancies	Phase1/2	61	Incyte Corporation	July 2020	Melanoma	Aug 2025	No results posted yet.
NCT03516981	A Study of Biomarker-Directed, Pembrolizumab (MK-3475) Based Combination Therapy for Advanced Non-Small Cell Lung Cancer (MK-3475-495/​KEYNOTE-495)	Phase2	318	Merck Sharp & Dohme LLC	October 2018	Advanced Non-Small Cell Lung Cancer	June 2025	No results posted yet.
NCT04931654	A Study to Assess the Safety and Efficacy of AZD7789 in Participants With Advanced or Metastatic Solid Cancer	Phase 1/2	232	AstraZeneca	September 2021	Advanced Solid Tumors	Aug 2026	No results posted yet.
NCT03946670	STIMULUS-MDS1: Sabatolimab + Hypomethylating Agents vs Placebo + HMAs in previously untreated higher-risk Myelodysplastic Syndromes	Phase 2	127	Novartis	Terminated Dec 2024	Myelodysplastic Syndromes	July 2024	Did not meet primary endpoints: CR 22% vs 18%; no PFS benefit.
NCT04266301	STIMULUS-MDS2: Sabatolimab + Azacitidine vs Placebo + Azacitidine in higher-risk MDS & CMML-2	Phase 3	530	Novartis	Discontinued February 2024	Myelodysplastic Syndromes/CMML-2	Oct 2024	Discontinued for futility; did not meet overall survival endpoint

*This information is available on https://clinicaltrials.gov/, (accessed on 6 July 2024).

## 7 Enhancing immunotherapy efficacy through strategic combinations

Recent studies have explored the potentiation of anti-PD-L1 and CD40-based immuno-chemotherapy combinations in heterogeneous pancreatic tumors through context-specific GITR agonism. Utilizing four representative immunocompetent mouse models, this research underscores the importance of GITR agonist therapy in enhancing the efficacy of existing immuno-chemotherapy strategies. This work provides a promising avenue for overcoming resistance mechanisms in pancreatic cancer, a malignancy known for its dismal prognosis (101).

Another significant contribution to GITR-focused preclinical trials is the study on the combination of PD1 inhibition and GITR agonism with fractionated stereotactic radiotherapy for treating recurrent glioblastoma. The combination of retifanlimab (anti-PD1) and INCAGN01876 (GITR agonist) may offer a novel therapeutic strategy for glioblastoma patients, suggesting an improvement in overcoming the high resistance of glioblastoma to conventional therapies (100).

Furthermore, some studies have focused on the heterogeneous cellular responses to GITR and TIGIT immunotherapy in the human gastrointestinal tumor microenvironment. Their findings emphasize the complexity of GITR’s role within the tumor microenvironment, suggesting that personalized approaches may be necessary to fully leverage the therapeutic potential of GITR agonism in gastrointestinal cancers (6).

## 8 Future perspectives in GI oncology: harnessing the potential of VISTA, TIGIT, GITR, STING and TIM-3

In the dynamic field of gastrointestinal (GI) cancer research, the emergence of innovative immunotherapy targets like VISTA, TIGIT, GITR, STING, and TIM-3 signals a groundbreaking shift in cancer care. This forward-looking perspective not only highlights the vast potential of these targets in scientific and clinical realms but also underlines the importance of adopting a holistic strategy to fully harness their advantages. Key to this endeavor is the leverage of advanced technologies, including next-generation sequencing and machine learning, to unveil novel targets and forecast treatment outcomes, facilitating the creation of efficient, tailored therapies.

### 8.1 Checkpoint modulation plus cellular or vaccine platforms

- Armoured CAR-T cells: CRISPR/Cas9 PD-1-knock-out mesothelin-CAR-T cells showed > 2-fold increased persistence and tumour clearance in gastric-cancer PDXs, and TIGIT-KO NK- or CAR-NK approaches further prevented fratricide (102).
- Checkpoint-enhanced cancer vaccines. Personalised neoantigen RNA vaccines combined with PD-1 and STING agonists elicited poly-epitope CD4^+^/CD8^+^ responses and durable regressions in refractory MSI-low colorectal cancer (103).
- Bispecific adaptor systems. A GITR-ligand/PD-1 bispecific adaptor fused into Claudin 18.2 CAR-T cells boosted IL-2 secretion and overcame the suppressive GI-TME *in vitro* and in murine orthotopic models (104).

These data suggest that coupling emerging checkpoints to cellular or vaccine platforms may convert transient responses into long-term disease control, a priority for hard-to-treat GI malignancies.

### 8.2 Prioritized combination strategies in the clinical pipeline

1) Dual TIGIT + PD-1 blockade in PD-L1-high oesophagogastric cancers, now yielding ORR ≈ 60% in phase II trials (88, 90). 2) STING agonists combined with PD-1 antibodies for microsatellite-stable colorectal and hepatocellular carcinomas to convert ‘cold’ tumours into inflamed phenotypes (105). 3) VISTA antagonism in myeloid-rich colorectal and pancreatic tumours where high VISTA expression drives immune escape (106). 4) GITR agonists paired with low-dose radiotherapy to expand effector CD8^+^ T cells while ablating intratumoural Tregs (107). 5) Bispecific TIM-3/PD-1 antibodies to forestall adaptive resistance seen with single-checkpoint inhibition (108). 6) Microbiome-modulating strategies (e.g., FMT) to amplify checkpoint efficacy across GI subtypes (109).

Actionable biomarkers should now extend beyond PD-L1 IHC. Multi-parameter panels already correlate with response: (i) tumour TIGIT-ligand (CD155) density together with TIGIT^+^CD8^+^ infiltration (101); (ii) VISTA-high MDSC score by CyTOF in colorectal cancer (109); (iii) circulating IFN-β-inducible chemokines (CXCL10, CCL5) as pharmacodynamic surrogates for STING agonism (105); (iv) baseline ctDNA mutation clearance at week 4 as an early on-treatment predictor of durable benefit. Prospective qualification of these signatures should be embedded in every registrational study.

A deep comprehension of the mechanisms behind therapy resistance is imperative for formulating approaches to maintain therapeutic efficacy. Customizing treatment regimens based on the patient’s genetic, molecular, and immune profiles will be a cornerstone in advancing personalized medicine within oncology.

Additionally, it’s paramount to assess the enduring safety and impact on the quality of life of these therapies, ensuring they are not only efficacious but also bearable for patients. Tackling regulatory and ethical issues, such as accessibility and fairness in treatment distribution, is crucial for the just dissemination of these innovations among varied patient demographics.

Key development hurdles remain. Species-specific differences in STING and TIM-3 epitopes complicate murine-to-human translation, delaying candidate selection. Dose-limiting cytokine-release with systemic STING agonists and myelitis seen with GITR agonism highlight the narrow therapeutic window. Tumour heterogeneity—the coexistence of immune-inflamed and immune-excluded niches within the same lesion—undermines single-biopsy biomarker reliability (110). Finally, real-world access is threatened by the high manufacturing cost of multi-antibody regimens and by region-specific reimbursement delays; modelling suggests a three-year lag between FDA approval and universal Canadian coverage (111). Addressing these issues will be as decisive as scientific progress itself.

Promoting international cooperation and the exchange of data among scientific and medical communities is essential for expediting discoveries and their clinical implementation, dismantling conventional obstacles and promoting a united effort to enhance patient care in GI oncology. This inclusive strategy, which considers technological, scientific, ethical, and social factors, lays the groundwork for revolutionary advancements in GI cancer treatment, offering improved outcomes and heralding an era where personalized medicine becomes the standard for patients worldwide.

## 9 Conclusion

Recent clinical and preclinical investigations of VISTA, TIGIT, GITR, STING, and TIM-3 in gastrointestinal oncology have yielded heterogeneous outcomes. While TIGIT and TIM-3 inhibitors demonstrated limited efficacy in randomized phase III trials (e.g., SKYSCRAPER-01, SKYSCRAPER-06, KeyVibe-008), early-phase studies of GITR agonists and STING agonists have shown manageable safety profiles but inconsistent antitumor activity. VISTA-targeting agents remain under evaluation, with no published efficacy readouts to date. Combination strategies—for example, dual TIGIT/PD-1 blockade or STING agonists plus PD-1 inhibitors—have generated higher response rates in select PD-L1–high and MSI-high cohorts but also highlight tumor-intrinsic resistance mechanisms in “cold” microsatellite-stable colorectal cancers.

Moving forward, rigorous biomarker-driven trial designs are essential for identifying patient subsets most likely to benefit, and the systematic incorporation of robust translational endpoints (e.g., on-treatment ctDNA clearance, STING pathway activation signatures) will guide adaptive treatment refinements. Realistic appraisal of dose-limiting toxicities and delivery hurdles—particularly for STING and GITR agents—must inform dosing and administration schedules. Ultimately, incremental advances grounded in controlled, evidence-based assessments will be necessary to transition these emerging checkpoints from early promise to reproducible clinical benefit in GI cancer.
